# Morphology and Behavior of the Early Stages of the Skipper, *Urbanus esmeraldus*, on *Urera baccifera*, an Ant—Visited Host Plant

**DOI:** 10.1673/031.012.5201

**Published:** 2012-04-12

**Authors:** Alice R. Moraes, Harold F. Greeney, Paulo S. Oliveira, Eduardo P. Barbosa, André V.L. Freitas

**Affiliations:** ^1^ Programa de Pós-Graduação em Ecologia, Universidade Estadual de Campinas, C.P. 6109, 13083-862 Campinas SP, Brazil; ^2^ Yanayacu Biological Station and Center for Creative Studies, Cosanga, Ecuador, c/o 721 Foch y Amazonas, Quito, Ecuador; ^3^ Departamento de Biologia Animal, Universidade Estadual de Campinas, C.P. 6109, 13083-862 Campinas SP, Brazil

**Keywords:** ant predation, Atlantic forest, butterfly, ejection behavior, fecal pellets, Hesperiidae, immature stages, leaf shelter, Urticaceae

## Abstract

The Neotropical genus *Urbanus* (Hübner) (Lepidoptera: Hesperiidae) contains around 34 described species, and is widely distributed from the extreme southern United States to Argentina. Here, we describe the larval morphology and behavior of *Urbanus esmeraldus* (Hübner)(Hesperiidae) in *Urera baccifera* (Urticaceae), a plant producing food rewards and fleshy fruits that attract ants (including predacious species) in a Brazilian forest. Larvae pass through five instars and construct two kinds of leaf shelters. Experiments with ejected fecal pellets showed that these can serve as cues to ground—dwelling ants that climb onto host plants and potentially attack the larvae. Manipulation with pellets placed at different distances suggests that ejection behavior decreases larval vulnerability to ant predation. Larval preference for mature leaves may be related with increased predation risk at ant—visited young leaves. The study shows that a combination of natural history and experimental data can help understand the life history of a butterfly using a plant with high predation risk.

## Introduction

Although studies on the biology and development of Neotropical Lepidoptera have received increased attention in recent years, the majority of moth families and butterfly families such as Hesperiidae, Lycaenidae, and Riodinidae (DeVries 1987, 1997) still lack general information. The full utility of information about immature biology and natural history—especially regarding its contribution to systematic studies of the Lepidoptera (Brown and Freitas 1994)—has yet to be recognized. Due to the lack of adequate material for immature comparison, and because adults are easier to collect and store (Scoble 1995), lepidopteran larval stages have not been as extensively researched as the subsequent adult stages. Lepidopteran classification has therefore relied mainly on studies of the adults, despite the fact that recent studies have repeatedly helped to resolve classifications based on immature morphology (Kitching 1985; Scoble 1995 and references therein; Freitas and Brown 2004; Willmott and Freitas 2006).

As lepidopteran larvae must avoid predation by a plethora of natural enemies such as bugs, spiders, scorpions, frogs, birds, marsupials, rodents, bats, and primates (Scoble 1995; Salazar and Whitman 2001), they exhibit a similarly diverse array of behavioral defenses. These defense strategies include hanging by silk threads, dropping from the host plant, feeding at night, biting, thrashing, removing frass from their vicinity, and building leaf shelters or frass chains (Brower 1984; Heads and Lawton 1985; Freitas and Oliveira 1992, 1996; Potting et al. 1999; Weiss 2003).

One of these, leaf shelter construction, is a behavioral defense strategy exhibited by members of at least 18 families of Lepidoptera. Larvae build external shelters on their host plants by folding, rolling, tying, or joining plant structures with silk (Scoble 1995). The architecture and complexity of shelters varies among species, but often involves a precisely executed series of cuts and folds, performed by larvae multiple times throughout their development (e.g., Greeney and Jones 2003; Weiss et al. 2004). Skipper butterflies (Hesperiidae) construct shelters throughout larval development and show a large amount of interspecific and ontogenetic variation, which may be phylogenetically informative within this group (Greeney and Jones 2003; Greeney 2009). While shelter building is ubiquitous among Neotropical skipper larvae (e.g., Moss 1949; Young 1985; Burns and Janzen 2001; Greeney 2009), the details of shelter architecture are available for only a few species (e.g., Greeney and Warren 2003, 2004, 2009; Weiss et al. 2004; Greeney and Young 2006; Greeney et al. 2010). Thus, like larval morphology, shelter architectural details remain unavailable for phylogenetic analyses for nearly all species of Hesperiidae.

**Figure 1.**
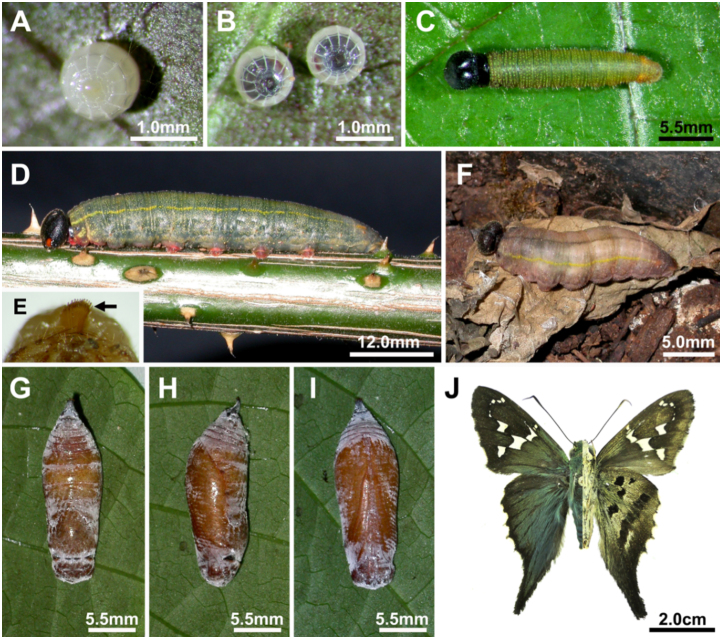
Developmental stages of *Urbanus esmeraldus*. (A) Upper view of an early egg; (B) upper view of a pair of eggs near hatching with dark head capsules of larvae visible; (C) dorsal view of second instar; (D) lateral view of a full growth fifth instar; (E) detail of last instar 10^th^ abdominal segment, the arrow showing the anal comb; (F) pre—pupa near pupation among dead leaves in the ground; (G, H, I) Pupa (dorsal, lateral, ventral); (J) adult male. High quality figures are available online.

The Neotropical genus *Urbanus* (Hübner) (Lepidoptera: Hesperiidae) contains around 34 described species (Mielke 2005). Host plant records are mostly in the Leguminosae and Poaceae (Kendall 1976; Cock 1986; Beccaloni et al. 2008), and some species are widespread and common pests of leguminous crops (Greene 1971; Dam and Wilde 1977; Nava and Parra 2002; Wendt and Carvalho 2006). Although *Urb. esmeraldus* (Butler) is among these (Wendt and Carvalho 2001), its early stages have never been described in detail and there is no information available of its larval shelter architecture. *Urbanus esmeraldus* (Figure 1J) is widely distributed from the extreme southern United States to Argentina (Mielke 2004), and is reported to feed on several species of Leguminosae, as well as *Urera* (Urticaceae) (Kendall 1976; Dutra et al. 2006; Beccaloni et al. 2008).

Here, we describe the larval morphology and shelter—building behavior of *Urb. esmeraldus* from larvae collected and reared on the nettle *Urera baccifera* (L.) in southeastern Brazil. This host plant produces food rewards in the form of pearl bodies and fleshy fruits that attract over 20 ant species, some of which may attack caterpillars and affect their survival (Machado and Freitas 2001; Dutra et al. 2006). Since the presence of frass is known to increase attacks on hesperiid caterpillars by predatory wasps (Weiss 2003), laboratory experiments were carried out to investigate whether throwing fecal pellets at great distances by *Urb. esmeraldus* larvae could act as a defensive strategy by decreasing ant visitation to the host plant.

## Materials and Methods

### Study area

All fieldwork was carried out in the Santa Genebra Forest Reserve, Campinas, São Paulo, southeast Brazil (22° 49′ S, 47° 06′ W). The reserve is predominantly covered by semi—deciduous mesophytic forest, is generally warm and wet, and has drier winters with rainier periods during the summer. Mean annual temperature is 21.6 °C and average rainfall is 1381 mm (Morellato and Leitão-Filho 1995). Small saplings of *Ure. baccifera* are commonly found along the main trail in the forest, about 1000 m long, and at the southern border of the reserve.

Leaves bearing eggs were brought to the laboratory, and larvae were reared individually in 500 mL plastic containers together with fresh *Ure. baccifera* leaves and a piece of toilet paper to absorb excess moisture. Containers were cleaned daily and leaves replenished whenever necessary (every two or three days). Data were taken on behavior and development times for all stages.

Larvae of *Urb. esmeraldus* were collected and reared in May and December 2005. To avoid artifacts of shelter construction under laboratory conditions, descriptions of larval shelters are based only on those built in the field. Shed head capsules were preserved and measured with a microscope fitted with an ocular micrometer. Egg size was measured as height and diameter. The larval head capsule size was measured as the distance between the two groups of stemmata. Immatures were preserved in Kahle's solution for studies of body chaetotaxy (1^st^ instar) and general morphology. A large quantity of larval fecal pellets was frozen for the experiments on frass ejection (see below).

Scanning electron microscopy (SEM) was conducted using a JEOL® JSM-5800 microscope (JEOL Ltd., www.jeol.com), and samples were prepared in accordance with the following protocol: Critical point dried in a Bal-tec® CPD030 Critical Point Dryer (www.precisionballs.com) and attached with double stick tape to aluminum stubs; gold/palladium coated with a Bal-tec® SCD050 Sputter Coater.

#### Field observations and experiments

**Infestation of *Urera baccifera* shrubs by lepidopteran larvae.** In order to discover if *Urb. esmeraldus* larvae use leaves of *Ure. baccifera* according to some sort of preference, every shrub that had leaf shelters already built by the caterpillars was recorded. Leaves were classified into three age categories according to characteristics such as brightness, coloration, leaf size, and proximity to the apical meristem. Young leaves were smaller than the others, dark green in color, brighter, and located closer to the apical meristem. Mature leaves were bigger than the young leaves, less dark, less bright, and further from the apical meristem. Old leaves were typically the same size as mature leaves (although they were sometimes smaller), more withered, and yellowish green in color (based on Letourneau 1983).

Based on this classification, the number of leaves of each age category and the total number of leaves were recorded for each plant. Additionally, the number of shelters built in each leaf category was recorded. If there was more than one shelter of different sizes in the same leaf, only one shelter was counted; early—instar larvae occasionally built consecutive shelters on the same leaf. Data were analyzed with a G test to check if *Urb. esmeraldus* larvae used young, mature, and old leaves in the same proportion that they occurred on plants. 31 plants possessing shelters of *Urb. esmeraldus* were recorded.

**Experiments with fecal pellets and ant visitation.** In order to document the fecal pellet throwing ability of larvae, one 4^th^ instar and one 5^th^ instar *Urb. esmeraldus* were reared on individual potted plants in the laboratory. Pots containing the plants were placed over white paper (80 × 168 cm) and the location of fallen pellets was marked with a pen. Pellet location was marked daily, as well as the horizontal location of the caterpillar on foliage with relation to the plant base, until the larva abandoned the plant prior to pupation.

To test whether presence of larval fecal pellets (∼2 mm diameter) on the ground could induce predatory ants to climb onto nearby host plants (see Del-Claro and Oliveira 1996), laboratory experiments were performed using “artificial plants“ made of a wooden stick (∼30 cm high) and a foam sphere on the top (∼10 cm diameter). Behavioral trials were performed using the ant *Camponotus crassus* Mayr (Hymenoptera: Formicinae), a common visitor of the host plant in the study area (Machado and Freitas 2001), and an efficient predator of caterpillars on foliage (Sendoya et al. 2009). Six ant colonies (30–50 workers) were reared in artificial nests consisting of test tubes measuring 2.2 cm diameter × 15 cm length, with water trapped behind a cotton plug. Each artificial ant nest was connected by a paper bridge to a plastic tray (40 × 20 cm) containing a single artificial plant at its center. In the two weeks before trials, ants walked freely between the nest and the plastic tray with the artificial plant. Ant colonies were fed daily on the foraging arena with 50% sucrose— water solution and termites, but were deprived of food for 48 hours before trials.

The first experimental series investigated whether the presence of fecal pellets of *Urb. esmeraldus* larvae nearby the artificial plant would induce ants to climb onto it. The flip of a coin determined if a filter paper next to the artificial plant would contain either fecal pellets (treatment; seven pellets) or similar—sized pellets made of black paper (control; seven balls). Experimental pellets were placed ∼3 cm from the plant base on a round filter paper (15 cm diameter) positioned under the base of the artificial plant. Observations started when the first ant stepped on the filter paper, after which the number of ants climbing onto the artificial plant was counted for 10 min. After every trial the filter paper was discarded and the artificial plant was cleaned with alcohol to eliminate possible cues left by the ants. Tests were performed using six captive ant colonies, and a total of 18 trials were performed for each experimental group. Each colony was tested only twice a day, with an interval of 90 min between trials.

The second experimental series investigated whether the spatial deposition pattern of fecal pellets on the ground would affect the rate of ant visitation to artificial plants. Experimental pellets were designated by the flip of a coin as treatment or control. In the treatment group, three pellets were placed on each of four pieces of filter paper (∼ 1.5 × 1.5 cm) located 30 cm from the base of the artificial plant. In the control group the pieces of filter paper containing the fecal pellets were placed 5 cm from the artificial plant. Four ant colonies were used to perform 20 trials with each group of experimental larval pellets. Observations started when the first ant stepped on a filter paper, after which the number of ants climbing onto the artificial plant was counted for 10 min.

## Results

### Description of immature stages

**Egg (Figures 1A–B; 3A–D).** Spherical with flat base, dull white, decorated with 13 vertical ribs and poorly defined horizontal ribs (only one well defined). One day before larval eclosion it became dark in the top (head capsule of the growing larva). Height and diameter 1.1 mm (n = 2). Females of *Urb. esmeraldus* can lay several eggs per plant, with eggs laid singly or in small groups of two. Eggs were found underneath mature leaves, usually near the leaf veins.

**First instar (Figures 3E–F; 4).** Head capsule width 0.68–0.70 mm (mean = 0.69 ± 0.01 mm, n = 4). Maximum body length: 8 mm. Head capsule black, rounded and smooth, without visible projections. Body light yellow after eclosion, becoming greener after feeding, when green gut content is visible; prothoracic shield dark brown, divided mid—dorsally by a narrow light brown line. Lenticles conspicuous and present subdorsally in T1, laterally in A1–A8 and sublaterally, adjacent to anal proleg in A10. Spiracles in T1 and A8 conspicuously larger than the remaining (A1 to A7). Legs light brown, prolegs light yellow. A conspicuous anal comb visible in A10 (Figure 3F). Anal plate same color as body. Body chaetotaxy (following Stehr 1987) is illustrated in Figure 4. Duration: 6–7 days (mean = 6.67 ± 0.58 days, n = 3).

**Second instar (Figure 1C).** Head capsule black; width 1.00–1.10 mm (mean = 1.05 ± 0.04 mm, n = 9). Maximum body length: 11 mm. Body brownish green with darker gut content visible; a pair of sublateral yellow spots visible on A8; dark protoracic shield more visible than in previous instar. Legs light brown, prolegs greenish yellow. Anal plate same color as body. Duration: 3–4 days (mean = 3.33 ± 0.58 days, n = 3).

**Third instar.** Head capsule width 1.54–1.78 mm (mean = 1.68 ± 0.09 mm, n = 10). Maximum body length: 17 mm. Head capsule black. Body dark green, less bright than the previous instar; a pair of pale, thin, poorly defined, spiracular stripes extend most of the length of the body; thoracic segments with a reddish cast ventrally; a pair of conspicuous yellow spots on A8; protoracic shield black and divided in two by a light brown line. Legs dark brown, prolegs the same color as the body. Duration: 3–7 days (mean = 5 ± 1.15 days, n = 7).

**Fourth instar.** Head capsule width 2.67–3.00 mm (mean = 2.79 ± 0.11 mm, n = 12). Maximum body length: 32 mm. Head capsule black with minute pale setae, with a pair of dull orange spots next to the stemmata. Body similar in coloration to previous instar but with lateral stripes more conspicuous, pale green to white; body covered with short, pale setae; a pair of conspicuous well-defined yellow spots on A8 and A10; prothoracic shield dark brown and divided mid—dorsally in two by a light brown line; male individuals bearing a pair of dark yellow spots visible below the cuticle between segments A5 and A6. Legs black, prolegs green. Duration: 5–8 days (mean = 6.25 ± 1.04 days, n = 8).

**Fifth (last) instar (Figures 1D–F).** Head capsule width 3.89–4.75 mm (mean = 4.32 ± 0.26 mm, n = 15). Maximum body length: 49 mm. Head capsule black with sparse, minute pale setae; a pair of well—defined bright dark orange spots next to the stemmata, give the appearance of large, pupil—less eyes. Body dark brownish green, with a conspicuous subdorsal yellow line from T2 to A7; thoracic segments with a reddish coloration in sublateral region and expanding ventrally; prothoracic shield black, divided mid—dorsally in two by a light brown region, extending to the lateral region until near the spiracle; a pair of conspicuous orange spots on A8 and A10; male individuals bearing a pair of orange spots visible below the cuticle between segments A5 and A6. Legs black, prolegs red. Anal plate dark brown. Anal comb visible in A10 (Figure 1E). Two or three days before pupation, the body became brownish purple (Figure 1F); lateral stripes fade and a mid—dorsal line of visible gut contents becomes more apparent. Larvae leave the host plant before pupation and pupate in the soil or leaf litter below the host plant. Duration: 9–13 days (mean = 11 ± 1.51 days, n = 8).

**Pupa (Figure 1G–I).** Length: 20–26 mm (mean = 22.33 ± 1.59 mm, n = 15). Entirely brown, robust, without projections; a white waxy flocculance covers the entire pupa. Duration: 8–10 days (mean = 8.91 ± 0.67 days, n = 23).

### Larval behavior

Larvae of all instars rested singly in leaf shelters, which change predictably in form during ontogeny. *Urbanus esmeraldus* builds two different kinds of shelters during its development (see below). Larvae of *Urb. esmeraldus* do not feed while inside their shelters, and move to another area of the leaf (or an adjacent leaf in later instars). Besides building shelters, *Urb. esmeraldus* exhibits other behavioral defense tactics, such as biting and, more rarely, regurgitating. Larvae of *Urb. esmeraldus* do not thrash, but move rather slowly and remain attached to a silk mat laid down on the surface of the leaf.

### Shelter building and occupation

**Figure 2.**
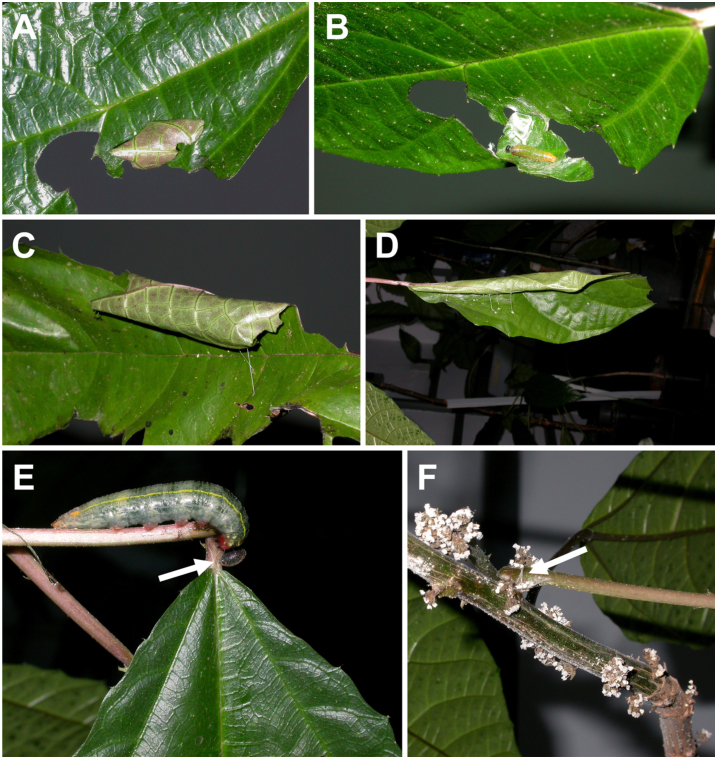
Behavior and structures of *Urbanus esmeraldus* larvae. (A, B) Shelter of second instar; in (B) the shelter was opened to show the position of the larvae inside it; (C) shelter of fourth instar; (D) shelter of last instar; (E) a last instar doing a second cut in the petiole of a mature leaf (arrow); (F) a detail of the incision made at the junction of the petiole with the stem. High quality figures are available online.

Young larvae (1^st^–3^rd^ instar) construct a peaked—roofed, cone—shaped or tent—like shelter by making two cuts from the leaf margin, folding the flap towards the center of the leaf and securing it to the surface with silk (Lind et al. 2001). They rest on the cone “ceiling” (Figures 2A–B). Larvae of 4^th^ and 5^th^ instars simply fold one side of the leaf big enough for them to rest underneath (Figures 2C–D). From the larvae reared on the plants in the laboratory, observations at night showed that fifth—instars totally (or almost totally) chewed out the petiole of the leaf where they build the shelter in two places, and deposited silk on the incisions. The leaf hung as if its petiole was broken and, a few days later, the larva made another incision above the first one, at the junction of the petiole with the stem, depositing silk on it. Then the leaf soon withered and died. Interestingly, the larvae also cut the petiole of the leaf on which they fed but left the leaf still hanging (Figures 2E–F).

**Larval infestation of *Urera baccifera* shrubs** *Urbanus esmeraldus* does not use different— aged leaves of *Ure. baccifera* in the same proportion as these occur on shrubs (Figure 5). No shelters at all were found on young leaves of any of the shrubs recorded, and most were recorded in mature leaves (31 out of 41 shelters). Although mature leaves are far more numerous, larvae still use the leaves of *Ure. baccifera* in a disproportionate manner (G = 13.72, d.f. = 2, *p* < 0.01).

### Larval fecal pellets and ant visitation to host plant

Larvae of *Urb. esmeraldus* threw fecal pellets at great distances from the plant base (up to 88.4 cm), generating a scattered deposition pattern around the trunk (Figure 6). Trials revealed that presence of fecal pellets next to the plant base induced increased numbers of ants climbing on the artificial plant compared to control paper balls (*t* = -3.3896, d.f. = 17, *p* < 0.01; see Figure 7A). In addition, experiments with fecal pellets placed at different distances revealed that artificial plants with pellets nearby attracted ants in greater numbers (Z = -2.4853, *p* < 0.05) and with higher frequency (G = 14.17, d.f. = 1, *p* < 0.01) than those from which pellets were deposited at a greater distance (Figure 7B, 7C).

## Discussion

The early stage morphology of *Urb. esmeraldus* is similar to other members of the genus (Greene 1971; Young 1985; Cock 1986), though few studies have examined most species of *Urbanus* caterpillars in detail. Similarly, the shelter building and frass ejection behaviors of *Urb. esmeraldus* closely match information available for congeners (Greene 1971; Young 1985; Greeney and Sheldon 2008). Larvae of the hesperiid *Epargyreus clarus* are known to undergo ontogenetic changes in leaf shelter construction; they build four different types of shelters during their development (Lind et al. 2001). It is very likely that *Urb. esmeraldus* also undergoes such ontogenetic changes. Actually, both kinds of shelters built by *Urb. esmeraldus* are very similar to two of the shelters built by *E. clarus* (two—cut fold and leaf roll, see Lind et al. 2001 for more details). Ontogenetic changes in shelter size and style may be explained by biological needs and/or physical capabilities of the larva, which change as it grows. As suggested by Lind et al. (2001), large larvae are able to manipulate large pieces of leaves and cutting may become unnecessary; 4^th^ and 5^th^ instars of *Urb. esmeraldus* in fact only fold the leaves.

Lepidopteran larvae are usually able to overcome ant attacks through a number of behavioral mechanisms (Heads and Lawton 1985; Freitas and Oliveira 1992, 1996; Oliveira and Freitas 2004; Sendoya et al. 2009). Larvae of *Urb. esmeraldus* also display such behaviors, including biting in response to disturbance. It has been demonstrated that behaviors such as biting or thrashing can significantly reduce parasitism rates (Potting et al. 1999). They also regurgitate, although rarely. Regurgitating in response to successive attacks is common among the Lepidoptera, and it is often associated with the presence of dissuasive substances in the regurgitated fluid (Brower 1984; Freitas and Oliveira 1992; Salazar and Whitman 2001; Oliveira and Freitas 2004).

Many lepidopteran species that build some kind of external shelter on their host plant (folding, rolling, or tying some of the plant's structures) also display frass ejection behavior (Weiss 2003). Based on direct reports of frass ejection and on the presence of associated anal structures (such as a sclerotized comb generally found in caterpillars that eject frass), it was determined that this behavior occurs in at least 17 lepidopteran families. Interestingly, within some families, shelter—building larvae eject their frass, whereas non—shelter— dwelling species generally do not (Scoble 1995; Weiss 2003). According to some authors, frass may act as a chemical and visual cue to natural enemies (Stamp and Wilkens 1993; Müller and Hilker 1999; Weiss 2003, 2006). In the case of the hesperiid *E. clarus*, its frass ejection behavior is positively related to defense against predation by the wasp *Polistes fuscatus*—wasps attacked significantly more larvae that were in close proximity to frass (Weiss 2003). Because proximity to its own frass is likely to make the larva vulnerable, we inferred that ejected frass near the base of the host plant could provide cues to potential predators like ants (similar to hemipteran exudates acting as chemical cues to ants; see Del-Claro and Oliveira 1996). The scattered distribution of the fecal pellets on the ground (away from the plant), as observed for 5^th^ instar of *Urb. esmeraldus*, could therefore make it difficult for ants to locate the plant hosting the larva. Indeed, the experiments demonstrated that presence of fecal pellets on the ground could induce ants to climb onto a nearby vertical structure (artificial plant). The experimental manipulation of fecal pellets placed at different distances suggests that ejection behavior by *Urb. esmeraldus* may in fact decrease larval vulnerability to ant predation on the host plant. Given that ants have been observed attacking *Urb. esmeraldus* larvae on foliage, and that larval infestation levels are higher on ant—excluded compared to ant— visited plants (Dutra et al. 2006), frass ejection away from the host plant probably plays an important role at reducing the risk of ant predation on leaves (see also Machado and Freitas 2001).

The behavior of chewing out the petiole of the leaf exhibited by 5^th^ -instar of *Urb. esmeraldus* is also reported for several species of grass feeding hesperiines (Greeney and Jones 2003; Greeney and Warren 2009) and at least one other Pyrginae (Greeney and Warren 2004). However, its purpose remains yet to be discovered. First, it could be interpreted as a strategy to eliminate some toxic compounds of the plant. The cut in the petiole can lead to the elimination of the plant's secondary compounds together with the sap and/or may cause the internal flux of those compounds to diminish (Dussourd 1993; Lewinsohn and Vasconcellos-Neto 2000). We do not know, however, if *Ure. baccifera* produces latex or other secondary compounds. Moreover, if this behavior were really shaped by such feeding constraints, it would be expected of caterpillars to display other means of avoiding toxic compounds throughout their development (Lewinsohn and Vasconcellos-Neto 2000). In addition, feeding constraints do not explain why they chew out the petiole of the leaf where they rest. We suggest that it may also be a defense mechanism against natural enemies. The cut petiole may deter crawling predators (such as ants) from reaching the surface of the leaf, thus minimizing caterpillar exposure (Freitas and Oliveira 1992, 1996; Oliveira and Freitas 2004). Caterpillars may also be less exposed to avian predators, which would not search for prey on withered leaves (Heinrich and Collins 1983). Birds are important predators of older larvae and pupae, whereas arthropods are probably more important predators of eggs and early larvae (Scoble 1995). Finally, vibrations may be the main stimuli used by parasitoids when their hosts are concealed feeders (Djemai et al. 2004), and an incision on the petiole could reduce the transmission of substrate—borne vibrations to other parts of the plant.

The results demonstrate a preference of the larvae of *Urb. esmeraldus* for leaves of specific developmental stage (i.e., mature leaves). The observed infestation pattern could be related to the nutritional quality of young leaves that may be nutritionally richer than older ones and provide the larva the resources for a faster development (Damman 1987). On the other hand, the infestation pattern observed could also be due to the presence of toxic compounds in the young leaves. Host plant chemistry mediates food choice by many species of herbivores, and is also well known to affect plant quality and to cause negative impacts on herbivores (see Ode 2006 and references therein). Toxic plant substances can deter feeding by herbivores and confer a great selective advantage to the plant if they are not easily metabolized by herbivores into nontoxic derivatives (McKey 1979). In some plant species, young leaves can contain greater concentrations of secondary compounds (e.g., alkaloids, tannins, cyanogenic glycosides) than mature ones (McKey 1979). However, we are not aware if this would be the case in *Urb. baccifera*, as no records of the presence of secondary compounds in this plant exist.

On the other hand, plants receiving protection by visiting ants normally offer food rewards on plant parts more vulnerable to herbivore attack (Rico-Gray and Oliveira 2007 and references therein). The host plant *Ure. baccifera* possesses two types of ant—attractants that are located at the apex of the branches—pearl bodies produced by the new leaves, and fleshy fruits (Dutra et al. 2006). Ants are present on the plants during most of the year, and harvesting of pearl bodies by ants is especially conspicuous when new leaves are produced and these food rewards accumulate on the leaf surface (Dutra et al. 2006). It is thus possible that preference for mature leaves by larvae of *Urb. esmeraldus* is related to the increased risk of predation by ants at the upper part of the plant crown, as suggested for other phytophagous insects on highly ant—visited plants (see Oliveira 1997; Silva and Oliveira 2010).

The current field and laboratory study with *Urb. esmeraldus* and its ant—visited host plant *Ure. baccifera* illustrates how the combination of natural history and experimental data can add to our understanding of immature biology, host plant use, shelter—building, sanitation and defensive behaviors, and herbivore—plant—ant interactions.

**Figure 3.**
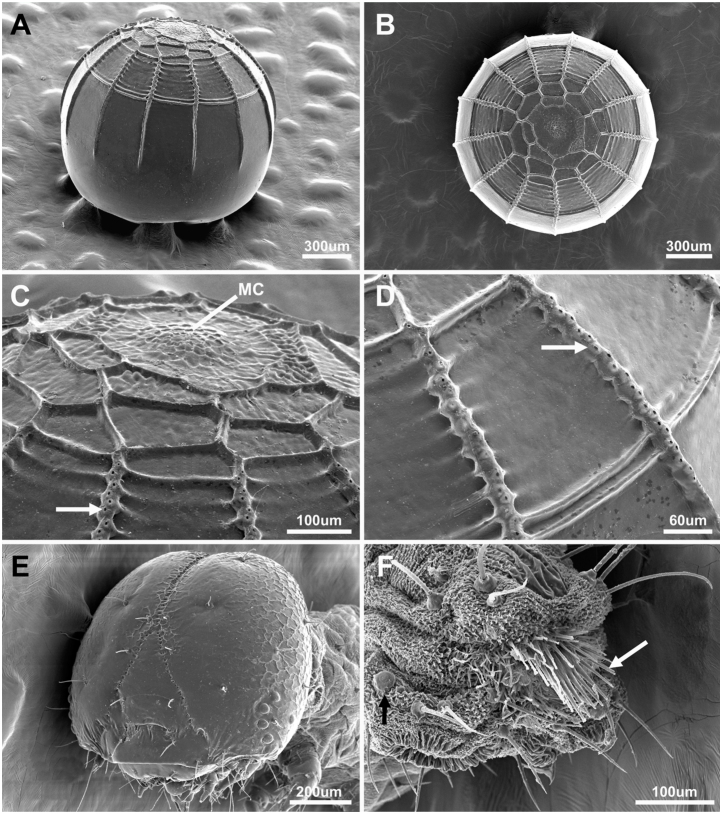
Early stages of *Urbanus esmeraldus*. (A, B) General view of the egg (lateral, dorsal); (C) detail of the egg tip, the arrow showing the micropilar region; (D) detail of the vertical rib of the egg, the arrow indicating the aeropiles; (E) general view of first instar head; (F) detail of first instar 10^th^ abdominal segment, the arrow indicating the anal comb. High quality figures are available online.

**Figure 4.**
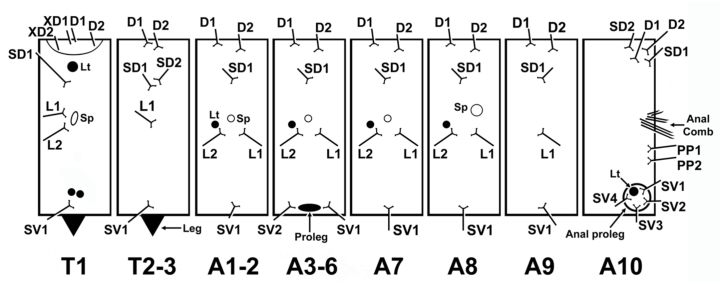
Chaetotaxy of first instar *Urbanus esmeraldus*. High quality figures are available online.

**Figure 5.**
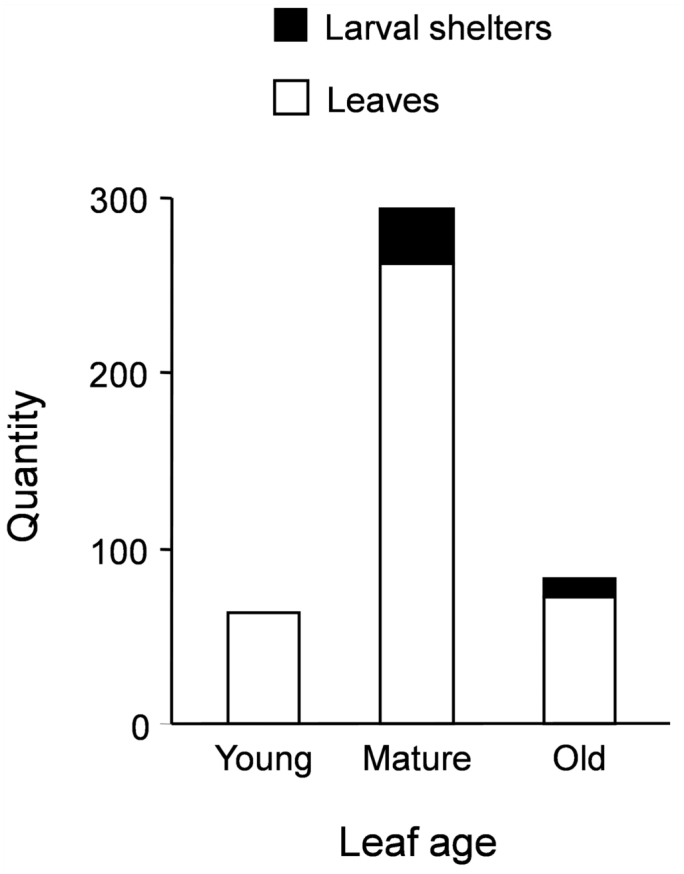
Distribution of larval shelters of *Urbanus esmeraldus* within the foliage of its host plant, *Urera baccifera*. Larval distribution among different— aged leaves differs significantly from their relative occurrence within the plant crown (G = 13.72, d.f. = 2, *p* < 0.01). High quality figures are available online.

**Figure 6.**
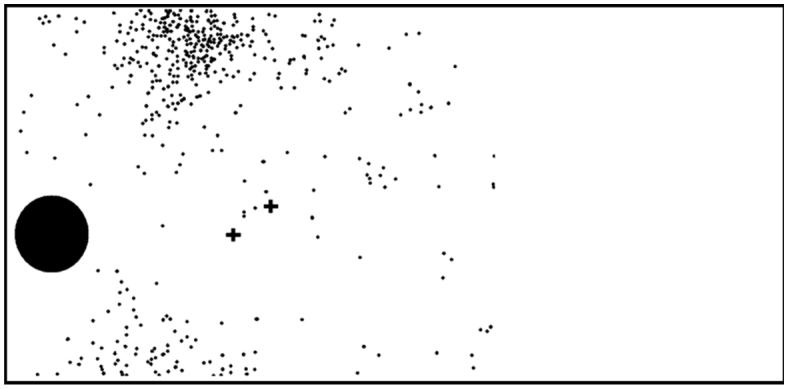
Spatial pattern of fecal pellets ejected by a last instar *Urbanus esmeraldus* feeding on a potted host plant (*Urera baccifera*) over five days in the laboratory. The two crosses represent the horizontal locations of the caterpillar on foliage (where it rested most of the time) with relation to the plant base (black circle). High quality figures are available online.

**Figure 7.**
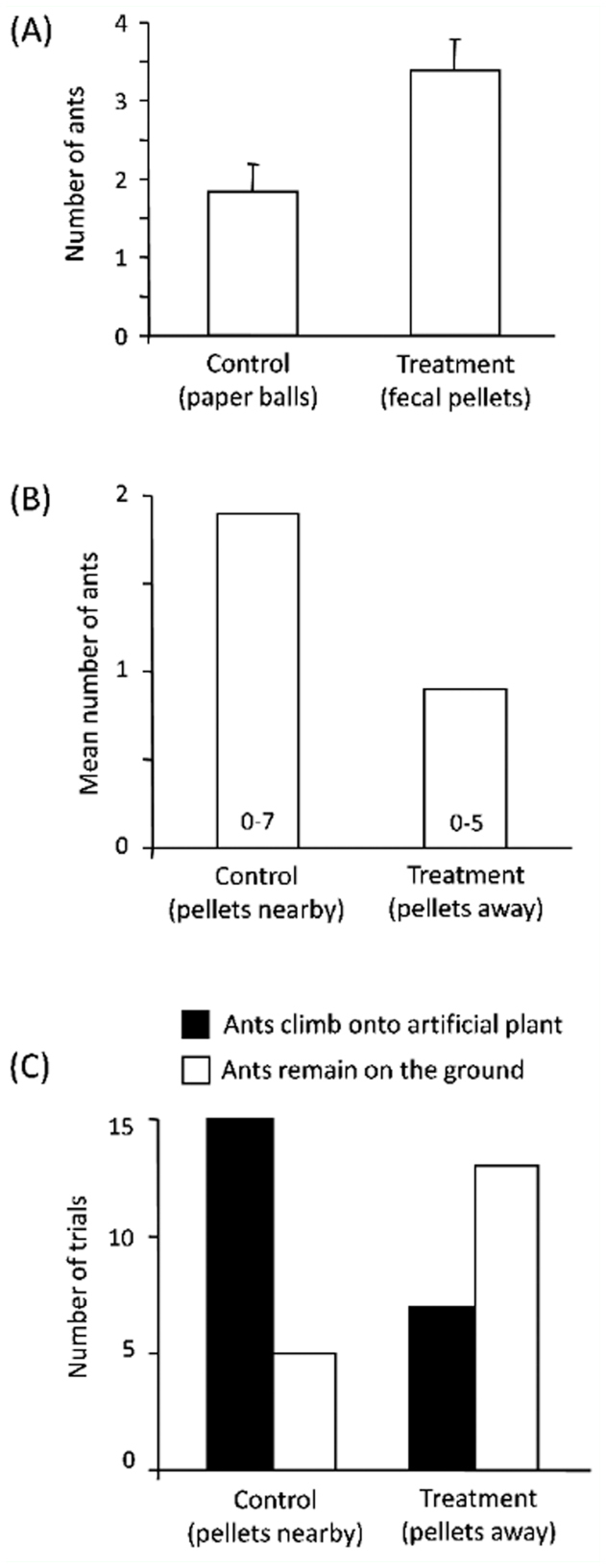
(A) Mean ± SE number of ants (*Camponotus crassus*) climbing onto an artificial plant after encountering nearby black paper balls and larval fecal pellets (*t* = -3.3896, d.f. = 17, *p* < 0.01). (B) Mean number of ants climbing onto an artificial plant after finding fecal pellets nearby (5 cm) or away (30 cm) from the plant base; numbers inside bars refer to range (Wilcoxon's test, Z = -2.4853, *p* < 0.05). (C) Proportion of trials in which ants climbed on an artificial plant after finding fecal pellets nearby or away from the plant base (G = 14.17, d.f. = 1, *p* < 0.01). High quality figures are available online.
